# Ectopic Adrenocorticotropic Hormone Syndrome in a Patient Diagnosed With Lung Adenocarcinoma and Hospitalized Due to Diabetic Ketonuria: A Case Report

**DOI:** 10.1155/crom/6307000

**Published:** 2026-07-29

**Authors:** Murat Ay, Erhan Bozkurt, Esra Özgül

**Affiliations:** ^1^ Department of Internal Medicine, Kütahya State Hospital, Kütahya, Turkey; ^2^ Department of Internal Medicine, Afyonkarahisar Health Sciences University, Afyonkarahisar, Turkey; ^3^ Department of Radiology, Afyonkarahisar Health Sciences University, Afyonkarahisar, Turkey

**Keywords:** diabetic ketonuria, ectopic ACTH syndrome, hypercortisolism, hypokalemia, lung adenocarcinoma, paraneoplastic endocrine syndrome

## Abstract

Ectopic adrenocorticotropic hormone (ACTH) syndrome (EAS) is most often a paraneoplastic manifestation of small‐cell lung cancer and is rare in lung adenocarcinoma. Its features include a cushingoid appearance, skin hyperpigmentation, muscle weakness, hypertension, glucose intolerance, hypokalemia, and metabolic alkalosis resulting from nonsuppressible, ACTH‐driven hypercortisolism. We report a 70‐year‐old man admitted with pneumonia and diabetic ketonuria without acidosis, in whom EAS secondary to a poorly differentiated lung adenocarcinoma was diagnosed. Key biochemical findings were a markedly elevated 8 a.m. plasma ACTH (149.3 pg/mL), grossly elevated serum cortisol (54.94 *μ*g/dL) with loss of diurnal rhythm, elevated 24‐h urinary free cortisol, and failure of cortisol suppression after both low‐ and high‐dose dexamethasone, with a normal pituitary MRI—a profile consistent with an ectopic ACTH source. What distinguishes this case is that the heralding presentation was a refractory metabolic emergency (hyperglycemia with diabetic ketonuria; HbA1c 6.3%, indicating no pre‐existing poorly controlled diabetes) rather than the classic cushingoid phenotype. The patient deteriorated rapidly and died on the third day after CT‐guided lung biopsy, before tumor‐directed therapy or confirmatory ACTH immunohistochemistry could be undertaken. This case highlights that EAS from lung adenocarcinoma may present primarily as a severe metabolic emergency and that early biochemical screening and control of hypercortisolism are critical.

**Key Clinical Message**

Ectopic adrenocorticotropic hormone (ACTH) syndrome should be considered in patients with hypokalemic metabolic alkalosis, hyperglycemia refractory to insulin infusion, and marked hyperpigmentation, even in the absence of a cushingoid phenotype. Early biochemical screening for hypercortisolism is essential in patients with refractory metabolic abnormalities because prompt recognition and control of hypercortisolism—pursued in parallel with tumor‐directed diagnosis—may improve outcomes. Ectopic ACTH syndrome is difficult to diagnose and requires a multidisciplinary approach.

## 1. Introduction

Ectopic adrenocorticotropic hormone (ACTH) syndrome (EAS) accounts for a minority of cases of ACTH‐dependent Cushing syndrome and is most commonly caused by small‐cell lung carcinoma and bronchial carcinoid tumors; rarer sources include thymic tumors, anaplastic thyroid carcinoma, pancreatic islet‐cell carcinoma, and pheochromocytoma [1]. EAS secondary to lung adenocarcinoma is distinctly uncommon, with only isolated cases reported [2–8]. The diagnostic approach to, and clinical perspective on, EAS are summarized in Figure 1.

**Figure 1:**
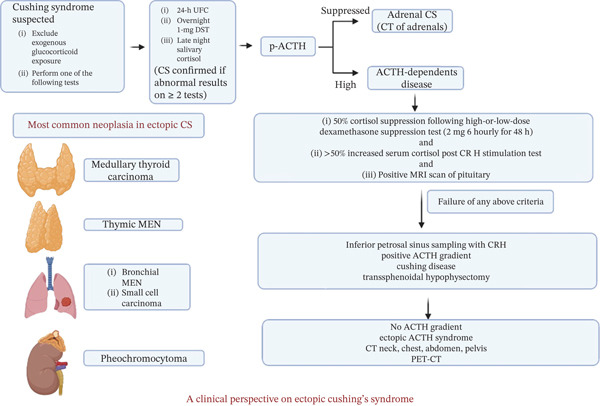
Clinical perspective on and diagnostic approach to ectopic adrenocorticotropic hormone (ACTH) syndrome (ectopic Cushing syndrome) summarizing the common etiologies, the biochemical confirmation of hypercortisolism, and the localization workup. Abbreviations: ACTH, adrenocorticotropic hormone; CRH, corticotropin‐releasing hormone; CS, Cushing syndrome; CT, computed tomography; DST, dexamethasone suppression test; MEN, multiple endocrine neoplasia; MRI, magnetic resonance imaging; PET‐CT, positron emission tomography–computed tomography; UFC, urinary free cortisol. Created with http://biorender.com/ (publication license/agreement number: MY297NOWZU).

Although EAS has occasionally been associated with colon, salivary gland, gallbladder, and prostate adenocarcinomas, lung adenocarcinoma remains a rare source [2–8]. In a focused PubMed search (terms including “ectopic ACTH,” “lung adenocarcinoma,” and “diabetic ketonuria/ketosis”), we did not identify a previously reported case in which EAS due to lung adenocarcinoma first manifested as hospitalization for diabetic ketonuria; to our knowledge, such a heralding presentation has not been emphasized. We therefore report this case to raise awareness that EAS may present primarily as a refractory metabolic emergency. Treatment of EAS depends on the underlying tumor and is discussed below.

## 2. Case History/Examination

A 70‐year‐old man with a history of hypertension, coronary artery disease, and prior coronary artery bypass grafting presented to the emergency department with cough, fever, tachypnea, and confusion. His regular medications were losartan, amlodipine, acetylsalicylic acid 100 mg, and atorvastatin 20 mg; he had no prior diagnosis of diabetes mellitus and no previous insulin use. Relatives reported new‐onset, progressive skin hyperpigmentation over the preceding 2 months. The physical examination was organized as follows. His general condition is as follows: drowsy, with limited orientation and cooperation, responding only partially to painful stimuli, and with signs of dehydration (reduced skin turgor and tonus, dry oral mucosa). Vital signs are as follows: blood pressure 125/85 mmHg, pulse 108 beats/min with occasional premature ventricular contractions, respiratory rate 26 breaths/min, and temperature 37.2°C. Skin findings are as follws: diffuse hyperpigmentation; facial appearance and adipose‐tissue distribution were not overtly cushingoid. Respiratory findings are as follows: rales in the right upper zone and diminished breath sounds in the right middle zone. Cardiovascular finding is as follow: irregular pulse with extrasystoles and no murmur. Neurological status is as follows: confusion without focal neurological deficit.

Chest computed tomography (CT) was performed to investigate the causes of the cough, fever, and rales using a Toshiba Aquilion (80 × 2) scanner (Otawara, Japan). CT revealed a solid lesion measuring approximately 43 × 35 mm in the posterior segment of the upper lobe of the right lung. Obstructive pneumonia was observed in the apical and posterior segments of the right upper lobe due to bronchial obstruction by the lesion (Figure 2A,B). Axial and coronal images also demonstrated a nodular lesion measuring 21 × 17 mm in the left adrenal gland corpus (Figure 3).

**Figure 2:**
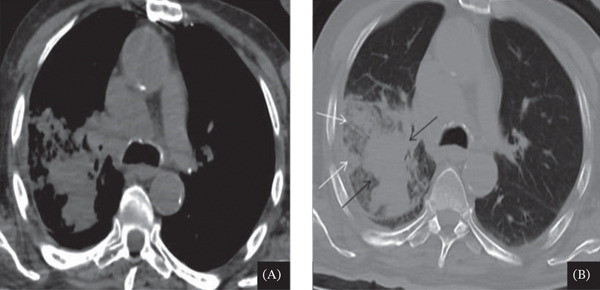
Axial chest computed tomography images in (A) mediastinal‐window and (B) parenchymal‐window settings demonstrate a solid mass (black arrows) in the posterior segment of the right upper lobe and concomitant obstructive pneumonia (white arrows).

**Figure 3:**
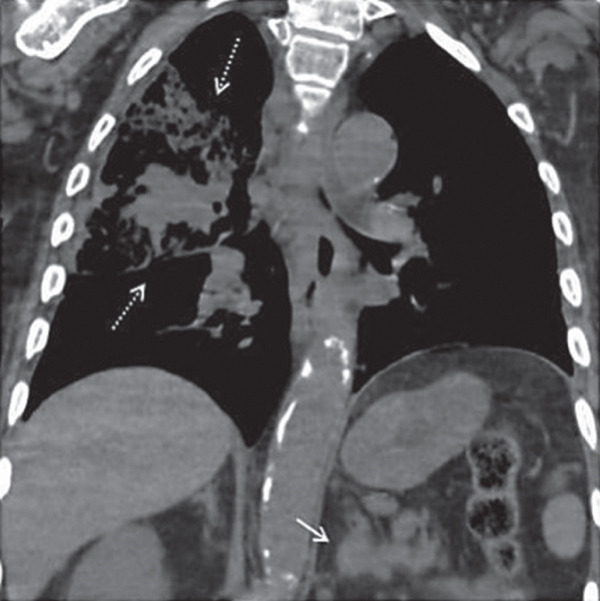
Coronal chest computed tomography image shows a nodular lesion in the left adrenal gland corpus (white arrows); the right upper‐lobe lung mass and associated obstructive pneumonia are also visible (dashed arrows).

Initial laboratory testing showed blood glucose 405 mg/dL, urea 72.36 mg/dL, creatinine 1.61 mg/dL, sodium 146 mmol/L, potassium 3.1 mmol/L, chloride 108 mmol/L, and C‐reactive protein 28.88 mg/L (reference < 5 mg/L). HbA1c was 6.3%, indicating the absence of long‐standing poorly controlled diabetes, and serum magnesium was normal (2.0 mg/dL). The leukocyte count was 19.75 × 10^9^/L (neutrophils 18.06 × 10^9^/L, lymphocytes 0.56 × 10^9^/L), hemoglobin 14.1 g/dL, and platelets 185 × 10^9^/L. Arterial blood gas analysis demonstrated alkalemia (pH 7.50, pCO_2_ 32.1 mmHg, pO_2_ 76.8 mmHg, HCO_3_^−26.1^ mmol/L), and the calculated serum anion gap was normal (11.9 mmol/L). Urinalysis showed 3+ glycosuria and 4+ ketonuria. Therefore, despite ketonuria and severe hyperglycemia, the patient had ketosis without acidosis rather than diabetic ketoacidosis; the predominant acid–base disturbance was alkalemia, consistent with cortisol‐driven hypokalemic metabolic alkalosis superimposed on tachypnea‐related respiratory alkalosis. Blood/serum ketone (beta‐hydroxybutyrate) measurement was not available at our center. The initial laboratory analyses are presented in Table 1.

**Table 1:** Analyses of the tests performed in the initial evaluation of the patient.

Laboratory parameters	Conclusion	Normal range
WBC	19.75	4.45–10.95 × 10^9^/L
Hemoglobin	14.1	13.3–17.5 g/dL
Neutrophil	18.06	×10^9^/L
Lymphocyte	0.56	×10^9^/L
Monocyte	2.21	×10^9^/L
PLT	185	162–367 × 10^9^/L
CRP	28.88	< 5 mg/L
Glucose	405	< 126 mg/dL
Sedimentation	74	< Age/2 (mm/h)
Ferritin	364	30–400 ng/mL
Urea	72.36	10–20 mg/dL
Creatinine	1.61	0.7–1.2 mg/dL
Sodium	146	135–145 mmol/L
Potassium	3.1	3.5–5.0 mmol/L
AST	42	< 40 U/L
ALT	25	< 41 U/L
Albumin	2.56	3.5–5.2 g/dL
Globulin	3.28	2.0–3.5 g/dL
T. bilirubin	1.42	< 0.31 mg/dL
D. bilirubin	1.12	< 1.2 mg/dL
ALP	404	< 190 U/L
INR	0.9	0.8–1.2
d‐dimer	154	< 500 ng/mL
Fibrinogen	400	193–412 mg/dL
pH	7.50	7.35–7.45
pCO_2_	32.1	32–48 mmHg
pO_2_	76.8	83–108 mmHg
HCO_3_^-^	26.1	22–29 mmol/L
Lactate	2.3	0.5–1.6 mmol/L
Urine analysis	3+ glycosuria, 4+ ketonuria	
Hepatitis and viral serology	Negative	
HbA1c	6.3	4.0%–5.6%
Chloride	108	98–107 mmol/L
Magnesium	2.0	1.7–2.2 mg/dL
Anion gap	11.9	8–12 mmol/L

Abbreviations: ALP, alkaline phosphatase; .ALT, alanine aminotransferase; AST, aspartate aminotransferase; HCO_3_^−^, bicarbonate; INR, international normalized ratio; pCO_2_, partial pressure of carbon dioxide; pH, hydrogen ion concentration; PLT, platelet; pO_2_, partial pressure of oxygen; WBC, white blood cell.

## 3. Differential Diagnosis, Investigations, and Treatment

The patient was admitted with diabetic ketonuria and obstructive pneumonia, and intravenous piperacillin–tazobactam and a temporary insulin infusion (for ketonuria) were initiated. Despite the insulin infusion, blood glucose persistently remained above 250 mg/dL. Hypokalemia was refractory (potassium 2.9 mmol/L) despite replacement; because serum magnesium was normal (2.0 mg/dL), hypomagnesemia‐related refractory hypokalemia was excluded, and the ongoing renal potassium wasting was attributed to cortisol‐mediated mineralocorticoid‐receptor activation. Given persistent kaliuresis, potassium chloride up to 160 mEq/day (in divided doses via combined intravenous and oral routes, with serial daily serum‐electrolyte and ECG monitoring) was required to maintain normokalemia. Oral spironolactone 100 mg twice daily was added for blood‐pressure control and to counteract the mineralocorticoid effect of cortisol excess.

Because the hypokalemia (potassium < 3.5 mmol/L) persisted despite replacement and the hyperglycemia (glucose > 200 mg/dL) persisted despite insulin infusion, screening for hypercortisolism was undertaken. The 8 a.m. plasma ACTH was 149.3 pg/mL (reference 7.2–63.3), 8 a.m. serum cortisol was 54.94 *μ*g/dL (reference 6.2–19.4), and 11 p.m. serum cortisol was 31.6 *μ*g/dL, indicating ACTH‐dependent hypercortisolism with loss of the normal diurnal rhythm. The 24‐h urinary free cortisol was markedly elevated (300 *μ*g/24 h). A low‐dose dexamethasone suppression test failed to suppress serum cortisol (36.2 *μ*g/dL), confirming endogenous Cushing syndrome.

## 4. Outcome and Follow‐Up

Pituitary magnetic resonance imaging (MRI) showed a normal gland without enlargement or erosion of the sella turcica (Figure 4), arguing against a pituitary source. A high‐dose dexamethasone suppression test was then performed (dexamethasone 8 mg orally between 11 p.m. and midnight); the following 8 a.m. plasma ACTH was 207.5 pg/mL and serum cortisol 31 *μ*g/dL, representing failure of adequate (> 50%) suppression and favoring an ectopic rather than a pituitary ACTH source.

**Figure 4:**
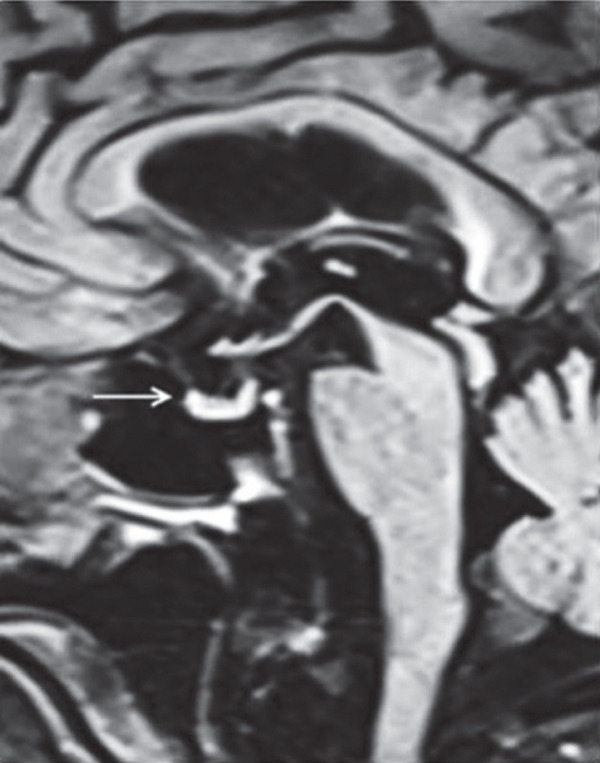
Sagittal FLAIR and postcontrast T1‐weighted pituitary magnetic resonance images show a normal pituitary gland (white arrow), without enlargement or erosion of the sella turcica, arguing against a pituitary source of ACTH.

A CT‐guided biopsy of the right upper‐lobe lung lesion was performed. On the third day after the procedure, the patient developed respiratory arrest and died. Histopathological examination revealed a poorly differentiated adenocarcinoma. Because of the patient′s death, confirmatory ACTH immunohistochemistry of the tumor, inferior petrosal sinus sampling, corticotropin‐releasing hormone (CRH) stimulation testing, and functional imaging (PET‐CT or somatostatin‐receptor imaging) could not be performed. The rapid clinical deterioration was likely compounded by the metabolic burden of severe hypercortisolism—refractory hyperglycemia, hypokalemia, and immune suppression predisposing to infection—underscoring the importance of early recognition and concurrent management of hypercortisolic complications alongside the diagnostic workup.

A consolidated clinical timeline, in accordance with the CARE guidelines, is presented in Table 2.

**Table 2:** Clinical timeline of the patient′s presentation, investigations, and outcome (CARE guideline).

Time point	Clinical events and findings	Investigations/interventions
~2 months before admission	Progressive skin hyperpigmentation (reported by relatives)	—
Day 0 (emergency department)	Cough, fever, tachypnea, confusion; obstructive pneumonia; glucose 405 mg/dL, K 3.1 mmol/L, 4+ ketonuria, alkalemia (pH 7.50)	Chest CT: 43 × 35 mm right upper‐lobe mass with obstructive pneumonia and a 21 × 17 mm left adrenal nodule; admission
Days 1–3 (ward)	Hyperglycemia > 250 mg/dL despite insulin; refractory hypokalemia (2.9 mmol/L); normal magnesium	IV piperacillin–tazobactam; insulin infusion; KCl up to 160 mEq/day; spironolactone 100 mg twice daily
During admission	Biochemical confirmation of ACTH‐dependent hypercortisolism	ACTH 149.3 pg/mL; cortisol 54.94 *μ*g/dL (loss of rhythm); 24‐h UFC 300 *μ*g/24 h; low‐dose DST nonsuppression (36.2 *μ*g/dL)
During admission	Localization workup; EAS favored	Pituitary MRI normal; high‐dose DST non‐suppression (ACTH 207.5, cortisol 31)
Biopsy day	Tissue diagnosis sought	CT‐guided biopsy of lung lesion
Biopsy +3 days	Respiratory arrest and death	Histopathology: poorly differentiated adenocarcinoma

## 5. Conclusion

In conclusion, EAS should be considered in patients with hypokalemic metabolic alkalosis, hyperglycemia refractory to insulin infusion, and marked hyperpigmentation, even when a cushingoid phenotype is absent. This case underscores the diagnostic difficulty of EAS and the need for a multidisciplinary approach to both diagnosis and management. Underlying causes of hypercortisolism must be actively investigated, and early recognition and prompt control of hypercortisolism are essential—pursued in parallel with tumor‐directed diagnosis—because delays in diagnosis and treatment may precipitate rapid and potentially fatal clinical deterioration.

## 6. Discussion

Paraneoplastic syndrome (PNS), caused by malignant diseases, is associated with clinical signs and symptoms elicited by hormones, immune cross‐reactivity, or cytokine production. PNS is not directly associated with primary or metastatic tumors [9]. Paraneoplastic endocrine syndromes are commonly detected in patients after the diagnosis of cancer. However, they may also be the first sign of an underlying malignancy, as in our case [10]. EAS is a paraneoplastic endocrine syndrome presenting with hypercortisolism, which is usually observed with carcinoids and small‐cell lung carcinoma and rarely with nonsmall‐cell lung carcinoma [11]. In our case, the patient was diagnosed with lung adenocarcinoma.

Lung adenocarcinoma presenting with PNS is rare [12]. In the literature, only a few cases of lung adenocarcinoma associated with EAS have been reported [8, 12–14]. Pan et al. [15] administered mifepristone and etoposide to a patient diagnosed with EAS and a thymic neuroendocrine tumor. However, surgical excision of the primary tumor was necessary to control the hypercortisolism and its complications. In EAS, hypokalemia may present complications ranging from mild muscle weakness to life‐threatening arrhythmias. Qiang et al. [16] have reported a case of EAS with rhabdomyolysis, in which the patient presented with profound hypokalemia and was diagnosed with small‐cell lung carcinoma. Kaplan et al. [17] emphasized the importance of methodological examination for hypercortisolism in cases of EAS and stated that a biochemical response was obtained with ketoconazole treatment without ectopic identification. They stated that treatment options for the control of hypercortisolism can be evaluated if an ectopic ACTH focus cannot be detected. Additionally, the combination of hyperglycemia, hypokalemia, metabolic alkalosis, and diabetic ketonuria should prompt consideration of hypercortisolism and be included in the differential diagnosis.

Although EAS arising from lung adenocarcinoma has been reported previously [8, 12–14], the distinguishing feature of the present case is its initial manifestation: rather than an evolved cushingoid phenotype, the patient first presented with a metabolic emergency—refractory hyperglycemia with diabetic ketonuria and hypokalemic alkalosis—as the heralding sign of an occult lung adenocarcinoma. The near‐normal HbA1c (6.3%) underscores that the metabolic derangement was acute and driven by hypercortisolism rather than long‐standing diabetes. Patients with EAS have a poor prognosis influenced by tumor histology, the presence of metastases, and the severity of hypercortisolism [18]; our patient exhibited marked hypercortisolism and died before tumor‐directed therapy could be planned after histopathological diagnosis. This presentation reinforces the need to screen for hypercortisolism in patients with disproportionate, refractory metabolic abnormalities.

The patient′s presentation can be unified mechanistically through cortisol excess. Glucocorticoid excess promotes hepatic gluconeogenesis and peripheral insulin resistance, producing hyperglycemia that is relatively refractory to insulin, as observed here; in the absence of pre‐existing diabetes (HbA1c 6.3%), the abrupt, severe hyperglycemia with ketonuria is best explained by acute hypercortisolism rather than chronic diabetes. At very high concentrations, cortisol overwhelms renal 11*β*‐hydroxysteroid dehydrogenase Type 2 and activates the mineralocorticoid receptor, causing renal potassium wasting, hypokalemia, and metabolic alkalosis—accounting for the hypokalemia that was refractory to replacement despite a normal serum magnesium. Cortisol‐induced immunosuppression predisposes to infection, consistent with the obstructive pneumonia at presentation, whereas protein catabolism and lipolysis complete the catabolic state.

Distinguishing EAS from pituitary Cushing disease is central to management. In this patient, markedly elevated ACTH with grossly elevated cortisol and loss of the diurnal rhythm confirmed ACTH‐dependent hypercortisolism, whereas failure of suppression after high‐dose dexamethasone together with a normal pituitary MRI favored an ectopic source. However, neither high‐dose dexamethasone testing nor pituitary imaging excludes pituitary disease with complete certainty; inferior petrosal sinus sampling—the diagnostic gold standard when imaging is inconclusive—could not be performed because the patient died, which limits definitive localization.

The differential diagnosis warrants emphasis. ACTH‐dependent causes include pituitary Cushing disease and ectopic ACTH secretion from small‐cell lung carcinoma, pulmonary or thymic carcinoid, medullary thyroid carcinoma, and other neuroendocrine tumors, as well as the rare ectopic CRH syndrome. ACTH‐independent adrenal causes (adrenal adenoma, carcinoma, or hyperplasia) were considered in view of the left adrenal nodule but were inconsistent with the markedly elevated ACTH. Primary hyperaldosteronism can cause hypokalemic alkalosis with hypertension but would not explain the elevated ACTH, the hypercortisolism, or the hyperglycemia. Stress hyperglycemia and diabetic ketoacidosis were also considered but were inconsistent with the near‐normal HbA1c, the absence of acidosis (normal anion gap with alkalemia), and the biochemical evidence of hypercortisolism. The overall constellation was therefore most consistent with EAS.

The 21 × 17 mm left adrenal nodule also merited consideration. In the context of markedly elevated ACTH, such a nodule most likely represents ACTH‐driven adrenal hyperplasia or an incidentaloma rather than an autonomous cortisol‐secreting adenoma or a metastasis; however, dedicated adrenal characterization (adrenal‐protocol washout CT or MRI and biochemical phenotyping) and follow‐up imaging were precluded by the patient′s death, and no prior imaging was available for comparison.

This report has limitations. As a single case, it cannot establish causality. Confirmatory ACTH immunohistochemistry of the tumor, inferior petrosal sinus sampling, CRH stimulation testing, and functional imaging (PET‐CT and somatostatin‐receptor imaging) could not be performed because the patient died on the third day after biopsy; thus, although the biochemical and imaging profile strongly supports EAS from lung adenocarcinoma, direct tissue confirmation of tumoral ACTH production is lacking and pituitary disease cannot be excluded with absolute certainty. Blood/serum ketone (beta‐hydroxybutyrate) measurement was unavailable at our center. These constraints reflect the fulminant clinical course and should be considered when interpreting the findings.

## Author Contributions

M.A.: conceptualization, investigation, methodology, analysis, interpretation of data, writing—original draft preparation, and writing—review and editing. E.B.: discussion, writing, review, and editing. E.O.: figure editing, review, and editing.

## Funding

No funding was received for this manuscript.

## Disclosure

All authors have read and approved the final version of the manuscript. Murat Ay (corresponding author and manuscript guarantor) had full access to all of the data in this study and takes complete responsibility for the integrity of the data and the accuracy of the data analysis. The lead author (Murat Ay) affirms that this manuscript is an honest, accurate, and transparent account of the case being reported; that no important aspects of the case have been omitted; and that any discrepancies from the case as planned have been explained. No formal statistical analysis was performed. This is a descriptive single‐patient case report; quantitative laboratory values are presented together with their reference ranges, and no statistical software was used.

## Ethics Statement

All procedures involving human participants were approved by the ethical standards of the institutional and/or national research committee and conducted in accordance with the 1964 Declaration of Helsinki and its later amendments or comparable ethical standards.

## Consent

Written informed consent was obtained from the patient for the publication of this case report and accompanying images. A copy of the written consent form is available for review by the editor‐in‐chief of the journal.

## Conflicts of Interest

The authors declare no conflicts of interest.

## Data Availability

Data sharing not applicable to this article as no datasets were generated or analysed during the current study.
